# Natural weathering studies of oil palm trunk lumber (OPTL) green polymer composites enhanced with oil palm shell (OPS) nanoparticles

**DOI:** 10.1186/2193-1801-2-592

**Published:** 2013-11-06

**Authors:** Md Nazrul Islam, Rudi Dungani, HPS Abdul Khalil, M Siti Alwani, WO Wan Nadirah, H Mohammad Fizree

**Affiliations:** School of Industrial Technology, Universiti Sains Malaysia, 11800 Kragujevac, Penang Malaysia; School of Life Science, Khulna University, Khulna, 9208 Bangladesh; School of Life Sciences and Technology, Institut Teknologi Bandung, Gedung Labtex XI, Jalan Ganesha 10, Bandung, 40132 West Java Indonesia

**Keywords:** Impregnation, IR-spectra, SEM, Wave number, Weight loss, Phenol formaldehyde

## Abstract

In this study, a green composite was produced from Oil Palm Trunk Lumber (OPTL) by impregnating oil palm shell (OPS) nanoparticles with formaldehyde resin. The changes of physical, mechanical and morphological properties of the OPS nanoparticles impregnated OPTL as a result of natural weathering was investigated. The OPS fibres were ground with a ball-mill for producing nanoparticles before being mixed with the phenol formaldehyde (PF) resin at a concentration of 1, 3, 5 and 10% w/w basis and impregnated into the OPTL by vacuum-pressure method. The treated OPTL samples were exposed to natural weathering for the period of 6 and 12 months in West Java, Indonesia according to ASTM D1435-99 standard. Physical and mechanical tests were done for analyzing the changes in phenol formaldehyde-nanoparticles impregnated (PF-NPI) OPTL. FT-IR and SEM studies were done to analyze the morphological changes. The results showed that both exposure time of weathering and concentration of PF-NPI had significant impact on physical and mechanical properties of OPTL. The longer exposure of samples to weathering condition reduced the wave numbers during FT-IR test. However, all these physical, mechanical and morphological changes were significant when compared with the untreated samples or only PF impregnated samples. Thus, it can be concluded that PF-NP impregnation into OPTL improved the resistance against natural weathering and would pave the ground for improved products from OPTL for outdoor conditions.

## Introduction

Recently, plenty of oil palm trunk (OPT) and oil palm shell (OPS) as a lignocellulosic material is producing due to the increase of oil palm tree plantation (Lua and Guo 2001). This huge amount of lignocellulosic material is mostly considered as an agricultural waste. The shortage of timber supply in wood-based industries and the negative impact of the huge agricultural waste has drawn the attention of researchers to work on OPT (Abdul Khalil et al. 2010a) and OPS (Dagwa et al. 2012). However, the utilization of OPT and OPS has still not optimally done and has lower economic value. Numerous researches and development efforts have been undertaken to utilize the oil palm biomass like OPS for active charcoal (Arami-Niya et al. 2010), OPT for furniture (Abdul Khalil et al. 2012), and empty fruit bunches for pulping (Astimar et al. 2002).

The effort that led the use of OPT for zero waste; it is necessary to find out alternative measures that ensure the use of OPT inside buildings, lightweight construction materials and furniture. Impregnation of chemicals into OPT and its modification might be a way to do this. Thermosetting resin impregnation into wood was started in 1936 (Stamm and Seborg 1939) and continued until early twentieth century (Ryu et al. 1991). Impregnation of resin into non-wood specially into OPT has started in the recent years (Abdul Khalil et al. 2012; Bhat et al. 2010a). However, the synthetic resins and OPT experience photo-degradation upon exposure to water and sunlight, especially ultraviolet (UV) (Geburtig and Wachtendorf 2010). The photo-degradation of polymers originates from excited polymer-oxygen complexes, which are mainly produced by introducing catalyst residues, hydroperoxide groups, carbonyl groups, and double bonds during polymer manufacturing (Zou et al. 2008). It has been shown that lignin is the constituent of wood that is most likely to undergo photo-degradation, which leads to the radical induced depolymerization of lignin, hemicelluloses, and cellulose at wood surfaces (Ndiaye et al. 2008). Therefore, color fading, chalking, surface roughening, cracking, damage the wood microstructure and strength weakening of materials may caused by weathering, restricting treated OPTL to specific outdoor applications (Feist 1990). Evans et al. (1996) reported that depolymerization of lignin and cellulose caused by photo-oxidation and furthermore, degraded by physical and biological factors, and water. However, it has been reported that UV light cannot penetrate deeper than 75 μm though degradation occurs deeper than this in combination with other factors (Hon 2001). Therefore, the material climate determined by wood moisture content and temperature, and their dynamics (Gobakken and Lebow 2010).

The degradation mechanisms are very complex and are influenced by many factors, e.g., rain, solar radiation and temperature, and thus, difficult to improve the weather resistance properties of wood. However, modification of wood might improve the weather resistance of wood by reducing the oxidation reactions. Different chemical modification methods have been practiced to improve the weathering resistance of wood by blocking the hydroxyl groups of cell wall polymers (Macleod et al. 1995). It was found that impregnation of methyl methacrylate monomer followed by polymerization reduce the weathering effects (Feist 1990). It was also reported that the impregnation of nanoparticles into lumber improves the weathering resistance (Lei et al. 2010).

However, all these works were done with inorganic nanoparticles. Accordingly, it may be possible to improve the weather resistance by impregnation of organic nanoparticles into wood. To the best of our knowledge, no prior report has been made on the weathering resistance properties of organic nanoparticles impregnated lumber. Thus, the aim of this study is to demonstrate the effects of OPS nanoparticles impregnation with PF resin on the natural weathering properties of OPTL. Physical, mechanical, and morphological properties of OPS nanoparticles impregnated green OPTL polymer composites would be analyzed to find out the effects.

## Materials and methods

### Material preparation

Oil Palm Trunks (OPT) were collected from a local plantation of 30 years old from Western Indonesia. OPTs were sawn to produce samples having the dimension of 50 × 50 × 500 mm (radial, tangential and longitudinal, respectively). Only the inner part of the OPT having the density of 0.29 g cm^-3^ were used in the study. At least 180 samples were prepared for one experiment. The samples were kiln dried until the moisture content reached to 14% before impregnation.

Oil palm shells (OPS) were collected from a palm-oil processing mill in Banten, Indonesia in the form of chips. Nano sized particles were prepared from this OPS chips by high energy ball milling (Pulverisette, Fritsch, Germany) process for 30 hours with 170 rev min^-1^ rotation speed of the planet carrier.

Phenol formaldehyde (PF) resin was used to impregnate OPS nanoparticles into OPTL. The commercial grade PF resin was collected from the Palmolite Adhesive Company, Indonesia. The properties of the PF resin are shown in Table 1.

**Table 1:** **Properties of PF resin**

Resin properties	Value
Viscosity @ 25°C (poise)	2.27
Specific Gravity @ 25°C	1.200
Resin Content @135°C (%)	42.5
pH (meter/25°C)	12.45
Molecular weight	4000

### Impregnation with OPS nanoparticles

PF resin was prepared at high molecular weight with a concentration of 15% w/w. Exactly 1, 3, 5 and 10% w/w OPS nanoparticles having the size of 50 to 100 nm was added to that PF resin for getting different concentrations of PF-NPI. The mixtures (PF resin and OPS nanoparticles) were compounded using twin screw extruder (Haake Model Rheodrive 500). The mixture was perfectly incorporated into the chamber to begin the process of impregnation. The PF-NPI was impregnated into OPTL by vacuum-pressure method. An initial vacuum was created for 15 minutes at 3 bar followed by pressure at 7 bar for 60 minutes, and then a final vacuum was created at 3 bar for 10 minutes. Untreated samples (without nano particle impregnation) were used as control.

### Natural weathering test

The natural weathering test was done according to the ASTM D1435-99 standard. The samples, after impregnation of OPS nanoparticles with resin, were exposed to natural weathering for a period of 6 and 12 months from June 2012 to May 2013 at Bogor, West Java, Indonesia. Annual average temperature, relative humidity, UV intensity, rainfall and long radiation were 25.9°C, 81.7%, 856.5 cal m^-2^, 1,570 mm, and 67.2%, respectively in the experimental area. The area is situated 325 m above the mean sea level, and the experimental place was on the roof of a 15 m high building having no shadow from a neighboring obstruction. Samples were placed vertically on the roof and exposed to the various weathering factors, such as precipitation, sunlight, temperature, moisture and wind.

### Testing of the materials

The OPS nano particles were analyzed by Scanning Electron Microscope (SEM) model ZEISS (type EVO 50, Germany), Transmission Electron Microscope (TEM) with a Philips CM12 instrument, and Fourier Transform Infrared (FT-IR) model Nicolet Avatar 360 (USA) for their structure, size and functional groups, respectively. Weight loss (%) of the treated samples was calculated according to ASTM D 3345-74 standard.

The physical properties, i.e. water absorption (WA), volumetric swelling coefficient (S), anti-swelling efficiency (ASE) and density, were measured according to BS EN 317: 1993, BS EN 317: 1993, and BS EN 325: 1993, respectively. Tensile properties, i.e. tensile strength (TS), tensile modulus (TM), and elongation at break (EB), and flexural properties, i.e. flexural strength (FS) and flexural modulus (FM) were measured by using a Instron (Model 5582, UK) Universal Testing Machine according to ASTM D 638 and ASTM D 790 standard, respectively. Impact strength (IS) was measured according to ASTM D256-04 standard by using a Ray Ran Universal Impact Pendulum (CS-1370). There were at least five replications for each type of test.

### Data analysis

Univariate Analyses of variance (ANOVA) were done with linear models in a completely randomized design (CRD) by using SPSS version 16.0.

## Results and discussion

### Characterization of nano structured materials from OPS

**Figure 1:**
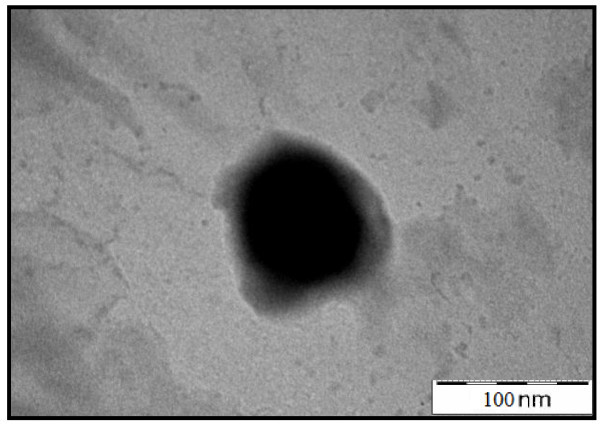
**TEM micrograph of OPS nanoparticles.**

**Figure 2:**
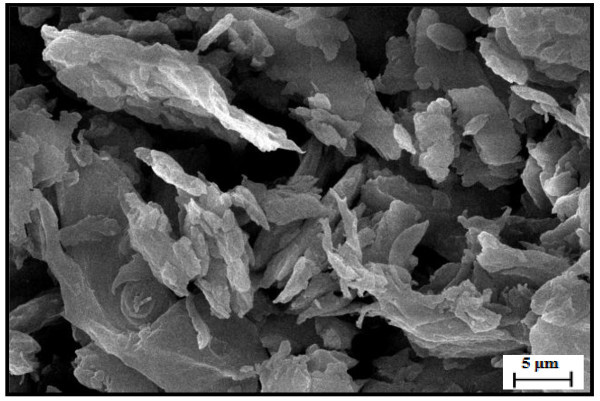
**SEM micrograph of OPS nanoparticles (1000× magnification).**

The microscopic investigation confirmed that the OPS particles transformed into nano-size particles. A TEM micrograph shows that the particle size ranges from 50 to 100 nm (Figure 1) with an average particle size close to 50.75 nm. The variation in particle size was developed during ball milling process. SEM micrograph revealed angular and irregular shape of OPS nanoparticles with crushing end (Figure 2). Paul et al. (2007) reported similar angular and irregular features for nano-structured materials derived from fly ash, though the fresh fly ash was mostly spherical in shape. They reported that the size reduction and all these irregularities in size and shape were evolved during high-energy ball milling.

**Figure 3:**
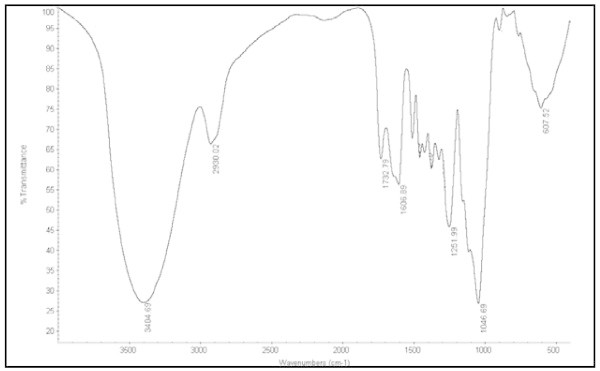
**FT-IR spectra of OPS nanoparticles.**

Figure 3 shows the FT-IR spectra of OPS nanoparticles. The OH stretch is usually broad and a strong absorption at 3404 cm^-1^ corresponds to the hydroxyl group (Afrouzi et al. 2013). The stretch at 2930 cm^-1^ is also strong and corresponds to -CH_2_^-^ bonds (Firoozian et al. 2011). In addition, the frequencies at around 1732 cm^-1^ and 1606 cm^-1^ corresponds to carbonyl (C = O) groups of hemicellulose (Colom et al. 2003), and the C-O and C = C bonds (Dagwa et al. 2012). The absorbance peak located at 1251 cm^-1^, 1046 cm^-1^ and 607 cm^-1^ are C-O stretching vibration in ethers (Wetzel et al. 1998), C-OH bonding (Robert et al. 2005), and stretching and bending of poly hydroxyl groups (Klinkaewnarong and Maensiri 2010), respectively.

### Change of weight due to natural weathering

Table 2 summarizes the average weight loss (%) and weight loss prevention ratio (%) of PF-NPI after 6 and 12 months of exposure. The PF-NP impregnation decreased the weight loss of PF-NPI OPTL due to weathering. The decline rate of this weight loss increased with the increase of nanoparticles percentage up to 5%. The weight loss also increased with the increase of exposure time. The average weight losses were 36.4%; 31.8%; 31.5%; 23.7%, and 27.5%, respectively for 0, 1, 3, 5, and 10% PF-NPI OPTL after 12 months of exposure at natural weathering condition. There were statistically significant difference between the nanoparticles concentration of 5% and other concentration (0, 1, 3, and 10%), and duration exposure of 6 and 12 months. However, there were no significant differences for 0, 1, 3 and 10% nanoparticles concentrations. On the other hand, the average weight losses of untreated OPTL were very high for both 6 (37.3%) and 12 (41.1%) months exposure time. It was found that there was significant difference between treated and untreated samples for any duration of exposure; however, this difference was not significant between different concentrations (C) of nanoparticles impregnation according to Duncan Multiple Range Test (DMRT). The duration of exposure (ET) also affected the weight loss significantly. However, the interaction between C and ET were not significant (Table 3).

**Table 2:** **Effect of OPS nanoparticles impregnation into OPTL on weight loss and weight loss prevention ratio after 6 and 12 months of weathering**

Nanoparticles (%)	Weight loss (%)		Weight loss prevention ratios (%)	
	6 months	12 months	6 months	12 months
OPTL	37.31 (0.89)*	41.09 (0.97)*	-	-
0	28.25 (0.79)Aa	36.37 (0.85) Ab	+24.28	+11.49
1	26.84 (0.82) Aa	31.84 (1.00) Ab	+4.94	+12.46
3	24.95 (0.95) Aa	31.47 (0.91) Ab	+16.68	+13.47
5	18.93 (0.86) Ba	25.59 (0.95) Bb	+32.99	+29.64
10	22.06 (1.03) Aa	29.51 (1.04) Ab	+21.25	+18.86

Values are means (n = 5); *Values in parentheses are standard deviation; different upper and lower case letters indicate significant differences at 95% confidence level.

**Table 3:** **A summary of the analysis of variance (p > 0.05) for concentration of nanoparticles and exposure time**

Variables	df	p- value										
		WL	WA	SC	ASE	D	TS	TM	EB	FS	FM	IS
Concentration (C)	4	0.543	0.000.	0.000	0.000	0.000	0.000	0.000	0.000	0.000	0.000	0.000
Exposure time (ET)	2	0.00	0.000	0.000	0.000	0.000	0.000	0.000	0.000	0.000	0.000	0.000
C × ET	8	0.294	0.016	0.000	0.063	0.000	0.936	0.562	0.155	0.997	0.759	0.295

WL: weight loss; WA: water absorption; SC: swelling coefficient; ASE: anti-swelling efficiency; D: density; TS: tensile strength; TM: tensile modulus; EB: elongation at break; FS: flexural strength; FM: flexural modulus; IS: impact strength.

Weight loss prevention ratios were higher in PF-NPI compared to the only PF impregnated OPTL. The weight loss prevention ratio (%) was the highest when there were 5% nano particle impregnation for both 6 and 12 months of exposure. From this result, it is clear that the rate of weight loss is the function of time. It indicates that weathering occurs due to photo-degradation of lignin in the materials, and leaching of those degraded lignin fragments from the exposed sample surfaces (Bhat et al. 2010b). The impregnated nanoparticles and PF resins also undergo the leaching process.

As reported earlier, weight loss of the exposed surface of the weathered specimens was normally due to the formation of water soluble products in addition with gaseous and volatile products (Futo 1974;1976). The exposed samples then attacked by the microbes, which also reduced the weight (Bhat et al. 2010b). However, weathering varies with many factors like species of wood, density, and climatic conditions (amount of irradiation, rain action, wind) (Feist 1990;1983).

### Change of functional groups due to natural weathering

**Figure 4:**
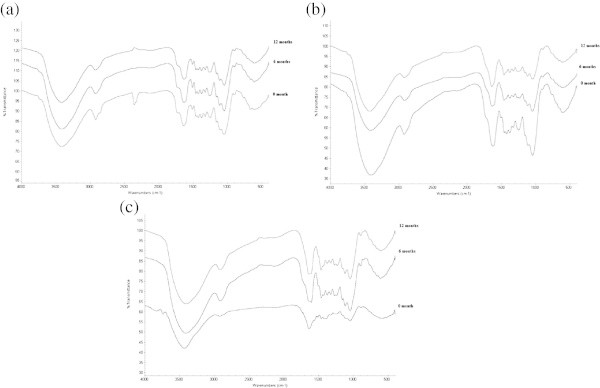
**FT-IR spectra of OPTL at different conditions. (a)** dried, **(b)** PF impregnated, and **(c)** PF-NPI OPTL.

Figure 4 shows the FT-IR spectra of dried OPTL, PF impregnated and PF-NP impregnated OPTL at 0, 6 and 12 months of exposure to the weathering condition. It was found that impregnation of PF or PF-NP caused some significant changes in the FT-IR spectra of the OPTL. The assignments of the characteristic IR absorption peaks in OPTL are listed in Table 4. The overall absorption peak decreased with the increase of exposure duration. A strong absorption peak was observed at 3419 cm^-1^, 3415 cm^-1^, and 3419 cm^-1^ for 0, 6, and 12 months exposure, respectively for the dried OPTL (Figure 4a). However, these peaks were are 3412 cm^-1^, 3414 cm^-1^ and 3423 cm^-1^ for PF, and at 3740 cm^-1^, 3412 cm^-1^ and 3413 cm^-1^ for PF-NPI OPTL, respectively for 0, 6, and 12 months exposure. The IR spectrum in the range of 3423–3412 cm^-1^ represent the stretching vibrations of O-H bond in cellulose (Pandey and Pitman 2003). However, the absorption peak appeared at 3740 cm^-1^ for PF-NPI at 0 month exposure was also assigned to hydrogen bond (O-H) stretching vibration (Blitz and Augustine 1994). The spectra of 1047–1045 cm^-1^ represents silicate minerals (Si-O bonds) (Georgokapoulos et al. 2003) which was not found in PF-NPI treated OPTL. The hydroxyl stretching bond of water (3435 cm^-1^) (Pongjanyakul et al. 2009) was only found in PF-NPI OPTL before the exposure to weathering condition. The peak for aromatic ring (C = C in plane) was only found at 1606 cm^-1^ for PF-NPI OPTL at 6 months exposure. The broad absorption band at around 1120 cm^-1^ attributes to a stretching vibration of Si-O-Si linkage (Galeener 1979).

**Table 4:** **Changes of FT-IR spectra due to the exposure to weathering condition at different exposure durations**

Treatment	Wave numbers (cm ^-1^ )			Assignments and Remarks
	0 month	6 months	12 months	
Dried OPTL	3419	3415	3419	stretching vibrations of O-H bond in cellulose (Pandey and Pitman 2003)
	2922	2925	2925	CH_2_ asymetry stretching (Pandey and Pitman 2003)
	2358	2360	-	2360-2358 (C = O stretching due to presence of carbondioxide) (Devi and Maji 2012)
	1641	1636	1639	- 1641 (amide (N-C = O) (Devi and Maji 2012)
				- 1636 (C = O, C = C) (Devi and Maji 2012)
				- 1639 (C = O, C = C) (Devi and Maji 2012)
	-	-	1253	1253 (Guaiacyl ring structure lignin) (Pandey and Pitman 2003)
	1047	1047	1046	1047-1046 (silicate minerals (Si-O bonds) (Georgokapoulos et al. 2003)
	608	608	613	presence of poly hydroxyl groups (Klinkaewnarong and Maensiri 2010)
PF impregnated	3412	3414	3423	stretching vibrations of O-H bond in cellulose (Pandey and Pitman 2003)
	2924	2919	2923	CH_2_ asymetry stretching (Pandey and Pitman 2003)
	1620	1640	1639	- 1620 (OH bending) (Devi and Maji 2012)
				- 1640 (amide (N-C = O) (Devi and Maji 2012)
				- 1639 (OH stretching linked water to cellulose) (Pandey and Pitman 2003)
	-	-	1462	1462 (C-H deformation and aromatic ring vibration) (Sun et al. 1999)
	1246	-	-	destruction of the guaiacyl units (Sun et al. 1999)
	1045	1046	1045	- 1045 (silicate minerals (Si-O bonds) (Georgokapoulos et al. 2003)
				- 1046 (silicate minerals (Si-O bonds) (Georgokapoulos et al. 2003)
	-	891	-	- (CH deformation in cellulose) (Pandey and Pitman 2003)
	608	610	606	poly hydroxy groups (Klinkaewnarong and Maensiri 2010)
PF-NP impregnated	3740	3412	3413	3412-3413 stretching vibrations of O-H bond in cellulose) (Pandey and Pitman 2003)
	3435	-	-	(N-H stretching) (Pongjanyakul et al. 2009)
	2925	2914	2921	- 3435 (N-H stretching) (Pongjanyakul et al. 2009)
				- 2925-2914 (CH_2_ asymetry stretching) (Pandey and Pitman 2003)
	1637	1606	1640	- 1637 (C = O, C = C) (Sun et al. 1999)
				- 1606 (aromatic skeleton vibration in lignin) (Sun et al. 1999)
				- 1640 (amide (N-C = O) (Devi and Maji 2012)
	-	-	1467	1467 (C-H deformations and aromatic ring vibrations) (Sun et al. 1999)
	-	1118	1120	- 1118 (Aromatic skeletal and C-O stretching) (Sun et al. 1999)
				- 1120 (stretching vibration of Si-O-Si linkage) (Galeener 1979)
	1045	1046	1046	1046-1045 (silicate minerals (Si-O bonds) (Georgokapoulos et al. 2003)
	-	-	894	(CH deformation in cellulose) (Pandey and Pitman 2003)
	-	613	610	- 613 (poly hydroxy groups) (Klinkaewnarong and Maensiri 2010)
				- 610 (poly hydroxy groups) (Klinkaewnarong and Maensiri 2010)
	589	-	-	The zbend of N_2_O (Klinkaewnarong and Maensiri 2010)

After exposure to weathering condition, various chemical reactions took place such as dehydration, hydrolysis, oxidation, decarboxylation, and transglycosylation resulting the changes in FT-IR spectra (Kocaefe et al. 2008). Photo-induced degradation of treated and untreated wood caused the main changes in the absorption intensity as were reported by Temiz et al. (2007). However, the intensity of the changes of these bands was related to the change of functional groups and chemical structure of the samples.

Several peaks in the stretching vibrations of O-H bond in cellulose at region (3419–3412) cm^-1^ in spectrum of samples, which were changed to peak at region (3415–3414 cm^-1^) and 3423–3413 after 6 and 12 months, respectively. These findings of decreased intensity at the peak with increasing exposure time were in consistent with the study carried out by Yildiz et al. (2011). They reported that weathering process caused more reduction in the range of 1720 to 1740 cm^-1^ (C = O stretching) than heat treatment at all treatment temperatures and durations, suggesting that there were decreasing photo-oxidation of wood surface after sunlight irradiation.

The absorption peak changes with the increase of nanoparticles concentration and duration of exposure. The PF-NPI OPTL had chemical changes in lignin and cellulose similar to that of acetylated wood as was studied by Feist et al. (1991). The study suggests that the observed reduction in weathering (weight loss) of PF-NPI OPTL may be a result of polymerization of both resin and nanoparticles. The free radical process may be disrupted during weathering when these components are polymer impregnated function as barrier and the weathering process is then retarded (Feist and Hon 1984).

### Change of mechanical properties due to natural weathering

As expected, mechanical properties (tensile, flexural, and impact strength) of all samples deteriorated due to the weathering effects and it was the highest for 12 months exposure duration. Table 5 shows the change of mechanical properties due to weathering for different duration of exposure. Tensile Strength (TS), Tensile Modulus (TM) and Elongation at Break (EB) of PF-NPI (5% nano particle) decreased from 4.8 to 11.1%, 23.7 to 43.0% and 16.4 to 24.5%, respectively when the exposure duration increased from 6 to 12 months. This change was 2.4 to 4.4%, 16.0 to 28.3% and 8.0–13.3%, respectively for Flexural Strength (FS), Flexural Modulus (FM) and Impact Strength (IS). The change of all these mechanical properties was significantly higher for untreated OPTL samples compared to the treated one. PF resin and the OPS nanoparticles filled the cell lumen to form a rigid cross-linked polymer which improved the strength and stiffness of the OPTL (Nur Izreen et al. 2011). Thus, treated samples had higher mechanical properties compared to the untreated one even after weathering. Nur Izreen et al. (2011) reported similar mechanical properties losses due to natural weathering. The statistical analysis showed that both C and ET had significant effect on the mechanical properties of OPTL after exposing to weathering condition, however, their interaction had no significant effect on the tested properties (Table 3).

**Table 5:** **Effects of OPS nanoparticles impregnation on mechanical and physical properties due to the exposure to weathering condition at different exposure durations**

Nanoparticles (%)	Tensile strength (MPa)/month			Tensile modulus (GPa)/month			Elongation at break (%)/month			Flexural strength (MPa)/month			Flexural modulus (GPa)/month		
	0	6	12	0	6	12	0	6	12	0	6	12	0	6	12
0	9.81Aa	8.22Ab	6.70Ac	2.67ABa	1.89ABEb	1.02ABc	7.83Aa	7.15Ab	6.40Ac	14.46Aab	13.49Abac	12.62Acb	4.35Aa	3.65AEb	2.95ABCDEc
	(0.14)*	(1/05)*	(0.90)*	(0.15)*	(0.27)*	(0.15)*	(0.35)*	(0.30)*	(0.32)*	(0.24)*	(0.90)*	(0.81)*	(0.17)*	(0.33)*	(0.32)*
		-16.21**	-31.70**		-29.21**	-61.80**		-8.68**	-18.26**		-6.71**	-12.72**		-16.09**	-32.18**
1	12.51Ba	11.44Bba	9.75Bc	2.85BAa	1.98BAEb	1.33BAEc	7.65Ba	7.19BEb	6.42BEc	29.35Bab	28.21BEbac	27.35BEcb	4.67Ba	3.94BCb	3.12BACDEc
	(0.27)*	(1.00)*	(1.00)*	(0.17)*	(0.32)*	(0.28)*	(0.37)*	(0.35)*	(0.34)*	(0.91)*	(0.84)*	(0.97)*	(0.18)*	(0.26)*	(0.29)*
		-8.55**	-22.06**		-30.53**	-53.33**		-6.01**	-16.08**		-3.88**	-6.81**		-15.63**	-33.19**
3	17.17Ca	15.57CEb	14.25CEc	3.25CEa	2.62CDb	2.10CDc	7.18Ca	6.32CDba	5.38CDc	33.51Ca	32.72Cba	31.68Cc	4.81Ca	3.89CBb	3.20CABDEc
	(0.11)*	(0.94)*	(0.96)*	(0.18)*	(0.36)*	(0.32)*	(0.38)*	(0.30)*	(0.30)*	(0.35)*	(0.68)*	(0.67)*	(0.19)*	(0.29)*	(0.29)*
		-9.32**	-17.01**		-19.38**	-35.38**		-11.98**	-25.07**		-2.36**	-10.78**		-19.13**	-33.47**
5	19.64 Da	18.69Dba	17.38Dc	3.51 Da	2.68DCb	2.00DCc	6.42 Da	5.37DCb	4.85DCc	38.55 Da	37.62Dbc	36.84Dcb	4.95 Da	4.16Db	3.55DACDEc
	(0.09)*	(0.95)*	(0.93)*	(0.20)*	(0.32)*	(0.32)*	(0.39)*	(0.31)*	(0.33)*	(0.23)*	(0.86)*	(0.67)*	(0.22)*	(0.30)*	(0.36)*
		-4.84**	-11.07**		-23.65**	-43.02**		-16.35**	-24.45**		-2.41**	-4.43**		-15.96**	-28.28**
10	16.72Ea	15.13EDb	13.50ECc	3.12ECa	2.12EABb	1.57EAc	7.35Ea	6.18EBb	5.28EBc	30.12Ea	28.76EBbc	28.02EBcb	4.57Ea	3.85EAb	3.19EABCDc
	(0.40)*	(0.83)*	(0.95)*	(0.32)*	(0.32)*	(0.30)	(0.33)*	(0.32)*	(0.34)*	(0.35)*	(0.80)*	(0.80)*	(0.27)*	(0.30)*	(0.36)*
		-9.51**	-19.26**		-32.05**	-49.68**		-15.92**	-28.16**		-4.52**	-6.97**		-15.75**	-30.20**
**Nanoparticles (%)**	**Impact strength (k.J/m** ^**2**^ **)/month**			**Density (g/cm** ^**3**^ **)/month**			**Water absorption (%)/month**			**Swelling coefficient (%)/month**			**Antiswelling efficiency (%)/month**		
	**0**	**6**	**12**	**0**	**6**	**12**	**0**	**6**	**12**	**0**	**6**	**12**	**0**	**6**	**12**
0	6.90Aa	5.76Ab	4.55Ac	0.42Aa	0.32Ab	0.20Ac	37.98Aa	45.69Aba	50.53Ac	6.36Aa	14.65Ab	20.04ACc	47.20Aa	43.27Ab	40.04Ac
	(0.28)*	(0.40)*	(0.41)*	(0.01)*	(0.03)*	(0.03)*	(0.93)*	(1.95)*	(1.52)*	(0.13)*	(1.94)*	(1.81)*	(1.32)*	(1.82)*	(1.84)*
		-16.52**	-34.06**		-23.81**	-52.38**		+16.87**	+24.84**		+54.59**	+68.26**		-8.33**	-15.17**
1	10.92BEa	10.00BEb	9.39BEc	0.66Ba	0.52Bb	0.35Bc	27.79Ba	37.48Bb	44.77Bc	5.75Ba	11.10BCEb	14.35Bc	56.23Ba	51.37Bb	46.35Bc
	(0.24)*	(0.41)*	(0.44)*	(0.01)*	(0.03)*	(0.03)*	(0.91)*	(1.51)*	(1.64)*	(0.17)*	(1.78)*	(2.08)*	(1.31)*	(1.75)	(1.99)*
		-16.52**	-34.06**		-21.21**	-46.97**		+25.85**	+37.93**		+48.20**	+59.19**		-8.64**	-17.57**
3	13.13Ca	12.23Cb	11.34Cc	0.70Ca	0.53CBb	0.41CEc	26.34CAa	34.61Cb	38.86Cc	4.81Ca	9.88CAEb	13.24CAEc	60.82Ca	57.39Cb	57.39Cc
	( 0.25)*	(0.33)*	(0.36)*	(0.01)*	(0.03)*	(0.03)*	(0.82)*	(1.67)*	(1.55)*	( 0.11)*	(1.93)*	(1.89)*	(1.44)*	(1.89)*	(1.89)*
		-6.85**	-13.63**		-24.29**	-41.43**		+23.89**	+32.22**		+51.31**	+63.67**		-5.64**	-13.76**
5	15.85 Da	14.58Db	13.75Dc	0.89 Da	0.72Dba	0.57Dc	24.15 Da	32.58DCb	40.61DCc	3.66 Da	6.91Dbc	8.67 Dcb	69.57 Da	62.48Db	57.97Dc
	(0.25)*	(0.42)*	(0.40)*	(0.02)*	(0.03)*	(0.04)*	(0.91)*	(1.65)*	(1.70)*	(0.10)*	(1.69)*	(1.85)*	(0.84)*	(1.72)*	(1.77)*
		-8.01**	-13.25**		-19.10**	-35.95**		+25.87**	+40.53**		+47.03**	+57.78**		-10.19**	-16.67**
10	11.00EBa	10.17EBb	9.25EBc	0.68EAa	0.49EBb	0.37EACc	27.20EBa	37.58EBb	43.55EBc	6.88Ea	9.37EACbc	11.40ECcb	43.14Ea	38.43Eb	31.11Ec
	(0.21)*	(0.40)*	(0.38)*	(0.01)*	(0.04)*	(0.04)*	(0.93)*	(1.85)*	(1.93)	(0.12)*	(1.93)*	(1.85)*	(1.44)*	(1.95)*	(1.99)*
		-7.54**	-15.91**		-27.94**	-45.59**		+27.82**	+37.54**		+26.57**	+39.65**		-10.92**	-27.88**

Values are means (n = 5); *Values in parentheses are standard deviation; **Changes of within weathering exposure (%); Different upper and lower case letters indicate significant differences at 95% confidence limit.

Several researchers have been proved that weathering reduced the mechanical properties (Bhat et al. 2010b; Esteves et al. 2008). They suggested that polymer degradation was mainly caused by chemical bond scission reactions in macro molecules. It was found that long-term exposed of the composites to elevated conditions affected the mechanical properties. Solar irradiance (UV component of the sunlight), relative humidity and temperature are the causal agents of this deterioration of natural fiber of impregnated samples (Lopez et al. 2006). The increase in the mechanical properties due to the chemical modification has been reported by several researchers. Bhat et al. (2010b) found that the flexural properties was attributed to the reaction of hydroxyl groups of cell wall polymers with the anhydrides, converting them into acetyl groups.

### Change of physical properties due to natural weathering

Similar to mechanical properties, physical properties also changed with the exposure time of PF and PF-NP impregnated OPTL. The change of these properties with different exposure time to weathering condition is shown in Table 5. The change of these physical properties was the lowest for PF-NPI followed by PF impregnation and untreated OPTL which indicated that treatment enhanced the properties of OPTL. The density of PF impregnated OPTL decreased 23.8 and 52.4% for 6 and 12 months of exposure to the weathering condition. The density change was positively correlated with the nanoparticles concentration, however, inversely correlated with the exposure time. The PF-NPI decreased the water absorption (WA) for a concentration of 5% nanoparticles; however, higher nanoparticles concentration increased the water absorption. This might be because of the lower degree of crystallinity of OPS nanoparticles which leaded to higher water absorption of the sample. The reduced degree of water absorption due to the replacement of the hydroxyl groups with carbon atoms in the PF chains has also been reported by several researchers (Lopez et al. 2006; Abdul Khalil et al. 2010b). Abdul Khalil et al. (2010b) found an interesting result that the highest water absorption because of the presence of more hydroxyl groups in the parenchyma tissue that enabled more hydrogen bonding formation. The swelling coefficient (SC) increased with the exposure time, however, decreased with the increase of nanoparticles concentration up to 5%. While, the antiswelling effeciency (ASE) decreased with the increase of exposure time. The ASE increases linear with increasing concentration nanoparticles at each exposure time. Accordingly, it can be states that PF-impregnated at various concentration nanoparticles and periods may prevent the rate of swelling resulting from decay. The PF-impregnation with 5% nanoparticles exhibited the lowest ASE change than PF-impregnation with 0, 1, 3 and 10% nanoparticles. The only PF-impregnation exhibited the highest ASE change. Thus, nanoparticles can be widely used to treat the PF-impregnated OPT for increasing the dimensional stability.

Based on these results, the PF resin and nanoparticles in OPTL reduced the porosity and minimized the physical properties change of OPTL resulting from weathering. Moreover, the formation of wall polymers inside the cell wall enhances the physical properties of the OPT (Abdul Khalil et al. 2010b). Statistical analysis indicated that concentration of nano particles as well as exposure time significantly affected the studied physical properties. The interaction of C and ET had significant effect on density, while no significant effect on WA and ASE (Table 3).

### Change of morphological properties due to natural weathering

**Figure 5:**
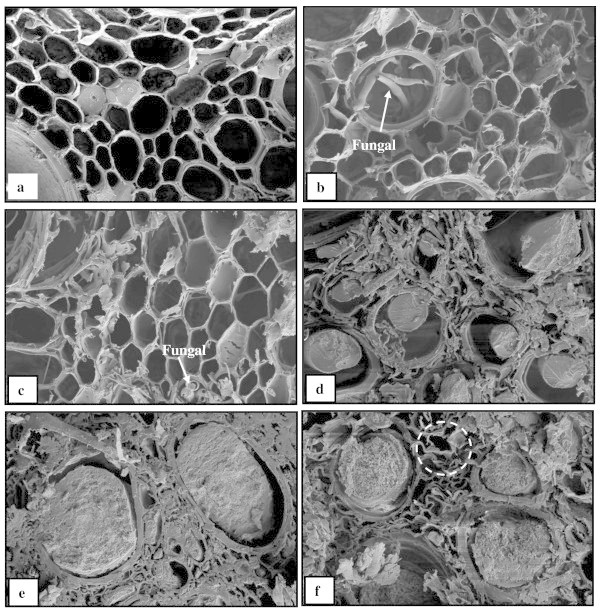
**SEM micrographs of OPTL at different weathering conditions. (a)** dried OPTL before weathering, **(b)** dried OPTL after 6 months weathering, **(c)** dried OPTL after 12 months weathering, **(d)** PF-impregnated OPTL before weathering, **(e)** PF-impregnated OPTL after 6 months weathering, and **(f)** PF-impregnated OPTL after 12 months weathering (500× magnification).

The OPT fibres showed great variability in size and shape i.e., both thick and thin cell wall as well as small and large lumina (Figure 5a). Reaction of OPTL due to natural weathering had significant effects on the changes of morphology. According to Feist and Hon (1984), absorption of UV light by lignin and photolysis and fragmentation of lignin resulting in the formation of aromatic and other radicals. These free radicals may then cause further degradation of lignin and photooxidation of cellulose and hemicelluloses. The phenomena can be well understood by comparing the SEM micrographs of OPTL cross-section before and after weathering (Figure 5a–5c).

Degradation of OPTL surfaces started at relatively low irradiation intensities having an fungi attack on the middle lamella whereas, higher intensities degraded the secondary cell walls (Fengel and Wegener 1989). The control sample showed loss of middle lamella, distortion of cell lumen and delamination of the cell wall after 6 months of exposure to weathering condition (Figure 5b). The OPTL cell wall was weathered and cell lumen became bigger than those of unweathered cell lumen which suggested the erosion of cell wall. Middle lamella appeared to be completely eliminated at the surface of OPTL after weathering with the presence of fungi in cell lumen of fiber. Similar findings were reported by Bhat et al. (2010b) where middle lamella showed holes, cell lumen were distorted and cell wall were degraded for *Acacia mangium* wood after 1 year of weathering.

On the other hand, distortion and erosion of fiber became more pronounced in the OPTL controls after 12 months of weathering (Figure 5c) particularly in the middle lamella and cell lumen. As mention earlier, fungi were found in the cell lumen after 12 months of weathering. Nevertheless, the middle lamella could still be clearly discerned in cell lumen of fiber after 6 months of weathering.

The changes of morphological properties of PF-NPI without nanoparticles after natural weathering are shown in Figure 5d–5f. The sample showed that the middle lamella could still be clearly discerned in these samples after 6 months of weathering (Figure 5e). Some defibrillation in the middle lamella and delamination in the cell wall was apparent in samples after 12 months of weathering (Figure 5f), but overall the changes were less pronounced than in OPTL impregnated samples. The effects of PF-impregnation could retard the formation of aromatic (lignin) radicals that initiate photo-oxidation. Alternatively, it is possible that resin matrix in OPTL scavenged free radicals preventing them from attacking lignin and cellulose. Such a suggestion is consistent with the observations by Nur Izreen et al. (2011) that benzoyl groups in wood obstruct free radicals and photostabilise polymers.

**Figure 6:**
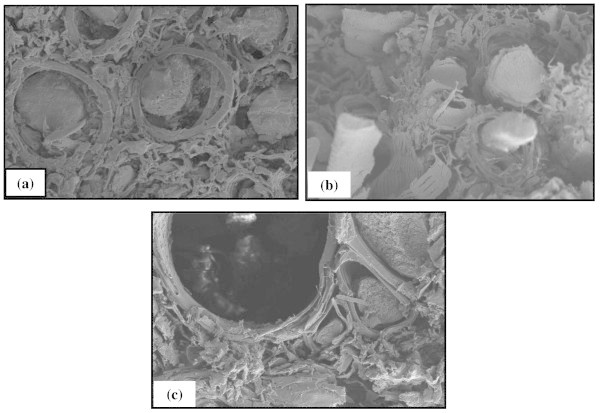
**SEM micrograph of OPTL at different weathering durations. (a)** PF-NPI before weathering, **(b)** PF-NPI after 6 months weathering, and **(c)** PF-NPI after 12 months weathering (500× magnification).

SEM micrograph suggested that the impregnated lumber with nanoparticles reduced the rate of weathering as removal of resin and nanoparticles from OPTL was needed first during weathering. Less weight losses of samples were also occurred due to this factor (Evans et al. 1996). Accordingly, the ability of PF impregnation with nanoparticles to protect lignin from photodegradation might explain why weight losses of impregnated OPTL during natural weathering were significantly lower than those of OPTL controls. Previous studies of the weathering of benzoylation of wood showed that benzoylation treatment reduced the formation of free radicals in wood when exposed to UV light, possibly because the benzoyl groups in wood absorbed UV light or scavenged free radicals (Esteves et al. 2008). The effects of water on OPTL weathering was also recognized as one of the principle causes of weathering by changing the dimension resulting cracks and checks formation and undergo degradation (Lopez et al. 2006). However, PF-NPI reduced the water uptake by OPTL during weathering and thus, reduced the degradation. Changes in morphology of PF-NPI specimens were also apparent after 6 months of weathering (Figure 6b). Degradation of the matrix occurred and splits developed in vessel cell (Figure 6b) after 6 months of weathering. After 1 year of exposure to the weathering condition, the matrix structure of PF-NPI specimens was fragile (Figure 6c) with further degradation of the cell walls and opened the cell lumens.

## Conclusions

Oil palm trunk lumber was successfully prepared by impregnation of phenol formaldehyde with OPS nanoparticles by vacuum-pressure method. The OPS nanoparticles appear to increase the quality of OPTL when it is exposed to natural weathering. Among all the PF-NPI OPTL, the addition of 5% nanoparticles exhibited superior physical and mechanical properties after 12 months of natural weathering. Degradation of the polymer matrix occurred for all PF-NPI during natural weathering, however, no significant differences were observed for the variation of concentrations of nanoparticles. Thus, matrix degradation was independent from the concentration of nanoparticles, however, dependent on the weathering duration. Thus, the impregnation of PF and OPS nanoparticles were effective in retarding the degradation of OPTL against the natural weathering.
